# Data on hollow NiO nanofibers prepared via electrospinning using camphene and subsequent various heat-treatment temperatures for anodes in lithium ion batteries

**DOI:** 10.1016/j.dib.2019.104074

**Published:** 2019-05-27

**Authors:** Jang Hyeok Oh, Jung Sang Cho

**Affiliations:** Department of Engineering Chemistry, Chungbuk National University, Chungbuk, 361-763, Republic of Korea

## Abstract

The data presented in this article are related to the research article entitled “New synthesis strategy for hollow NiO nanofibers with interstitial nanovoids prepared via electrospinning using camphene for anodes of lithium-ion batteries” [1]. Hollow NiO nanofibers were prepared by electrospinning process using camphene and subsequent heat-treatment process with various temperatures. The data presented in this manuscript showed the effect of the heat-treatment temperature of the as-spun fibers on the lithium ion storage properties of the hollow NiO nanofibers as anodes for lithium ion batteries. Each FE-SEM image, XRD pattern, cycle, and rate properties of the hollow NiO nanofibers obtained at various heat-treatment temperatures were investigated.

**Table:** Specifications Table

Subject area	*Chemistry*
More specific subject area	*Inorganic chemistry*
Type of data	*Figures*
How data was acquired	*FE-SEM, XRD, cycle and rate properties*
Data format	*Raw, analyzed data*
Experimental factors	*Heat-treatment temperature*
Experimental features	*Morphology, crystallite size of NiO, cycle, and rate properties*
Data source location	*Cheongju, Chungbuk, Republic of Korea*
Data accessibility	*Data included in this article*
Related research article	[1]*J. H. Oh, M. S. Jo, S. M. Jeong, C. Cho, Y. C. Kang, J. S. Cho, New Synthesis Strategy for Hollow NiO Nanofibers with Interstitial Nanovoids Prepared* via *Electrospinning Using Camphene for Anodes of Lithium-Ion Batteries.*https://doi.org/10.1016/j.jiec.2019.04.021

**Table:** 

**Value of the data** • Relevant data on the morphology and crystallite size of the hollow NiO nanofibers. • To investigate the effect of the heat-treatment temperature on the characteristics of the obtained nanofibers. • These data provide the importance of the heat-treatment temperature to obtain the hollow NiO nanofibers as anode materials.

## 1 Data

**Fig. 1:**
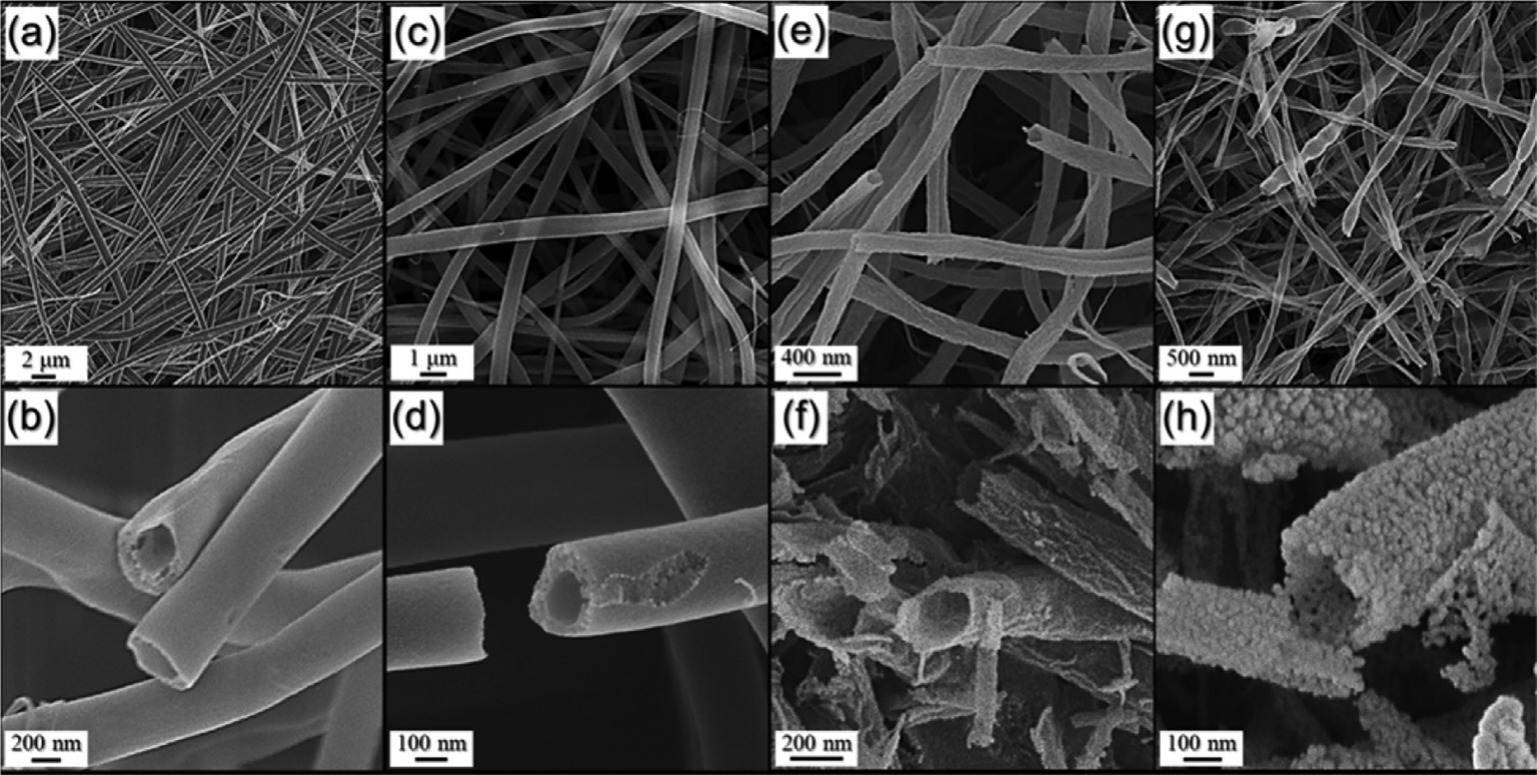
FE-SEM images of the samples: (a, b) as-spun fibers, (c, d) T-300, (e, f) T-350, and (g, h) T-400.

**Fig. 2:**
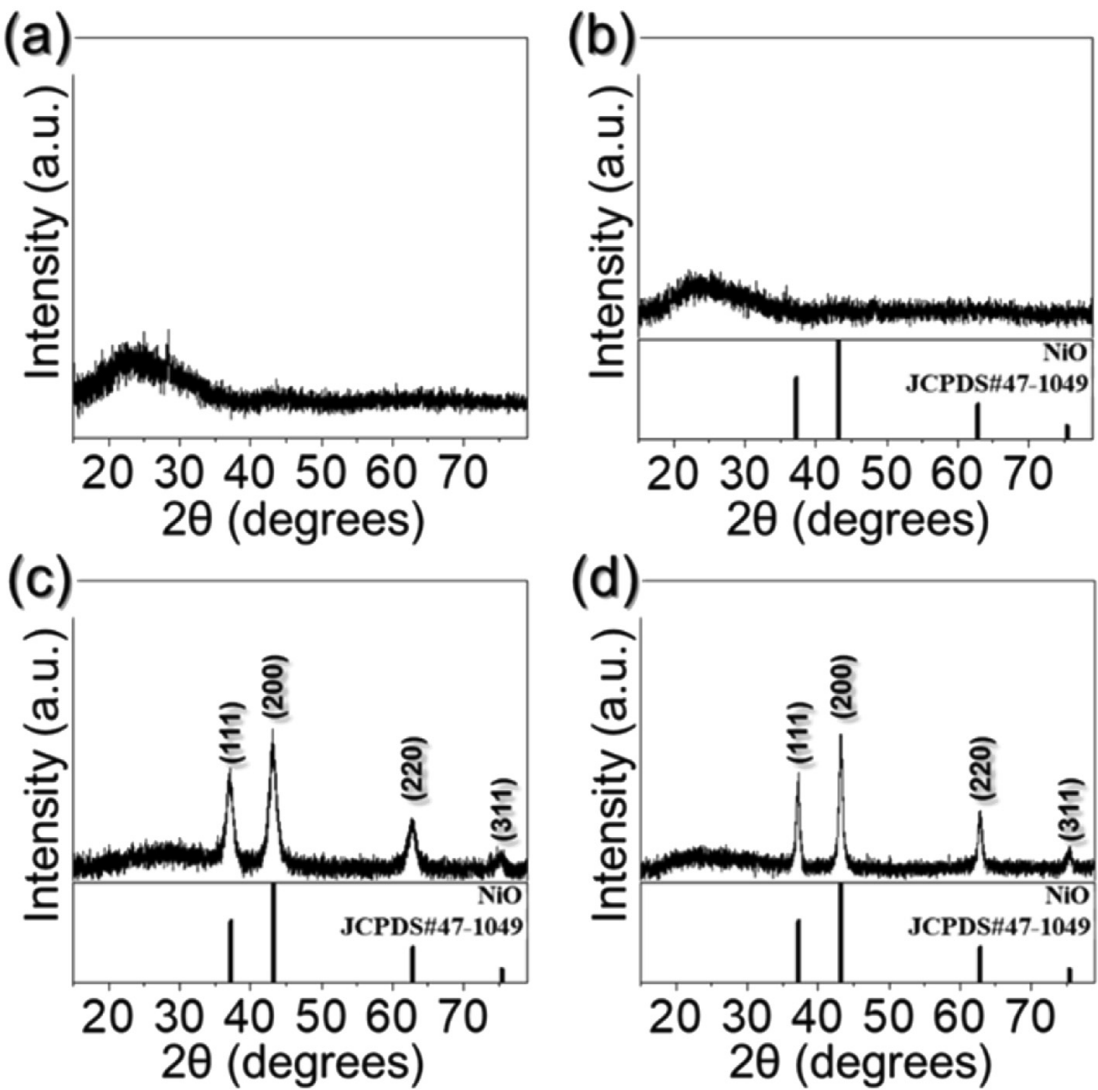
XRD patterns of the samples: (a) as-spun fibers, (b) T-300, (c) T-350, and (d) T-400.

**Fig. 3:**
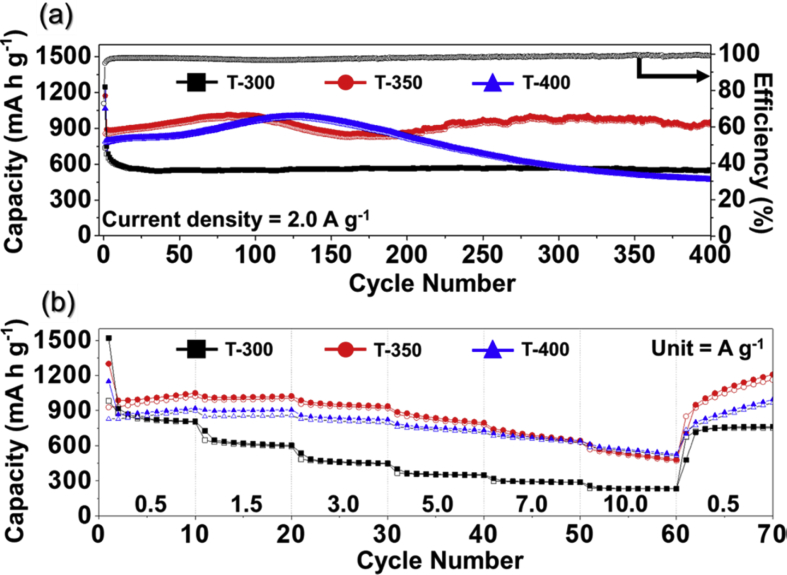
Lithium ion storage properties of the samples: (a) cycle and (b) rate performances.

The data presented in this study have been generated searching for a hollow NiO nanofibers as anode materials by electrospinning and subsequent heat-treatment process. Fig. 1 shows nanofiber morphologies obtained before and after heat-treatment with various temperatures. Fig. 2 shows the XRD patterns of nanofibers obtained before and after heat-treatment with various temperatures. Fig. 3 shows the electrochemical properties of the samples obtained by half-cell electrochemical test.

## 2 Experimental design, materials, and methods

Experimental details for the preparation of the hollow NiO nanofibers by electrospinning using camphene are written in reference [1]. Briefly, Nickel (II) nitrate hexahydrate, polyvinylpyrrolidone, acetic acid, and camphene were added in ethanol solvent to prepare a spinning solution. The solution was then electrospun. In order to investigate the effect of heat-treatment temperature of as-spun fibers on the morphology and crystallite size of NiO, the nanofibers were heat-treated at 300 °C, 350 °C, and 400 °C, respectively. These samples are denoted T-300, T-350, and T-400, respectively. The morphologies of as-spun nanofibers and the samples obtained after heat-treatments were shown in Fig. 1. The as-spun nanofibers exhibited a hollow inner space, even though no further processing was performed in Fig. 1a and b. As the heat-treatment temperature increased above from 300 to 350 °C, the shell thicknesses of the obtained nanofibers decreased in Fig. 1c–f. The shell of T-400 was observed to consist of nanocrystals with a mean size of 12 nm in Fig. 1h.

The phase and crystallite size of NiO in the samples obtained at different heat-treatment temperatures were shown in Fig. 2. The as-spun nanofibers and T-300 showed the amorphous-like phase in the XRD patterns (Fig. 2a and b). However, above heat-treatment temperature of 350 °C, cubic NiO phase was observed. The mean crystallite size of NiO nanocrystals comprising T-350 and T-400, calculated by Scherrer's equation to the (200) NiO peak were 6.1 and 12.3 nm, respectively.

The lithium ion storage properties of the samples obtained at different heat-treatment temperatures were shown in Fig. 3. The cycling properties of the samples at a current density of 2.0 A g^−1^ are shown in Fig. 3a. T-300 and T-350 showed better cycle properties compared to that of T-400. The initial discharge capacities of T-300, T-350, and T-400 were 1247, 1174, and 1066 mA h g^−1^, respectively, and their Coulombic efficiencies were 59.0, 72.9, and 74.0%, respectively. The discharge capacities of T-300, T-350, and T-400 after 400 cycles were 554, 956, and 471 mA h g^−1^, respectively. The rate properties of the samples are shown in Fig. 3b, with the current density increasing stepwise from 0.5 to 10.0 A g^−1^. The final discharge capacities of T-300 at 0.5, 1.5, 3.0, 5.0, 7.0, and 10.0 A g^−1^ were 807, 603, 450, 351, 290, and 233 mA h g^−1^, respectively. The final discharge capacities of T-350 at 0.5, 1.5, 3.0, 5.0, 7.0, and 10.0 A g^−1^ were 1050, 1024, 937, 796, 643, and 483 mA h g^−1^, respectively. The final discharge capacities of T-400 at 0.5, 1.5, 3.0, 5.0, 7.0, and 10.0 A g^−1^ were 921, 906, 824, 733, 642, and 528 mA h g^−1^, respectively.
